# A comparative screening of freshwater and marine microalgae for salinity-induced intracellular mannitol accumulation

**DOI:** 10.3389/fbioe.2026.1922237

**Published:** 2026-09-17

**Authors:** Shibin Nadersha, Ashfaq Ahmad, Syed Salman Ashraf, Jorge Rodríguez

**Affiliations:** 1 Department of Chemical and Petroleum Engineering, Khalifa University, Abu Dhabi, United Arab Emirates; 2 Research and Innovation Centre on CO_2_ and H_2_ (RICH), Khalifa University, Abu Dhabi, United Arab Emirates; 3 Department of Biological Sciences, Khalifa University, Abu Dhabi, United Arab Emirates; 4 Center for Biotechnology, Khalifa University, Abu Dhabi, United Arab Emirates

**Keywords:** industrial biotechnology, intracellular polyol, mannitol, microalgae, salinity stress response

## Abstract

**Introduction:**

The growing demand for sustainable, low-glycaemic sugar alternatives has increased interest in biological polyol production; however, comparative evaluations of microalgae for salinity-induced intracellular polyol accumulation remain limited.

**Methods:**

This study systematically screened two freshwater species (*Chlorella vulgaris* and *Scenedesmus obliquus*) and two marine species (*Dunaliella tertiolecta* and *Nannochloropsis oculata*) using a two-stage cultivation strategy to evaluate intracellular polyol accumulation and associated physiological responses under salinity stress. Cultures were grown under optimal conditions before exposure to moderate and high salinity levels (16 and 32 g L^−1^ for freshwater species; 60 and 80 g L^−1^ for marine species). Growth, nitrate assimilation, intracellular polyols, carbohydrates, proteins, chlorophylls, and carotenoids were quantified.

**Results:**

No detectable intracellular polyols were observed in the freshwater species under any treatment. In contrast, intracellular mannitol accumulated exclusively in the marine species, reaching maximum concentrations of 28 mg g^−1^ DW in *D. tertiolecta* and 34 mg g^−1^ DW in *N. oculata* under moderate salinity. Moderate salinity promoted the highest intracellular mannitol accumulation in both marine species. Although biomass-specific mannitol yields decreased under high salinity, volumetric mannitol concentration remained relatively stable in *N. oculata* but declined markedly in *D. tertiolecta*. Salinity stress also induced distinct species-specific changes in carbohydrate, protein, and pigment composition, as well as nitrogen assimilation.

**Discussion:**

These findings demonstrate that intracellular mannitol accumulation is a species-specific response to salinity stress rather than a universal microalgal adaptation. The comparative screening identifies *D. tertiolecta* and *N. oculata* as promising candidates for future optimization of stress-induced intracellular mannitol accumulation and provides a framework for strain selection in sustainable microalgal biotechnology.

## Graphical Abstract

**Figure:**
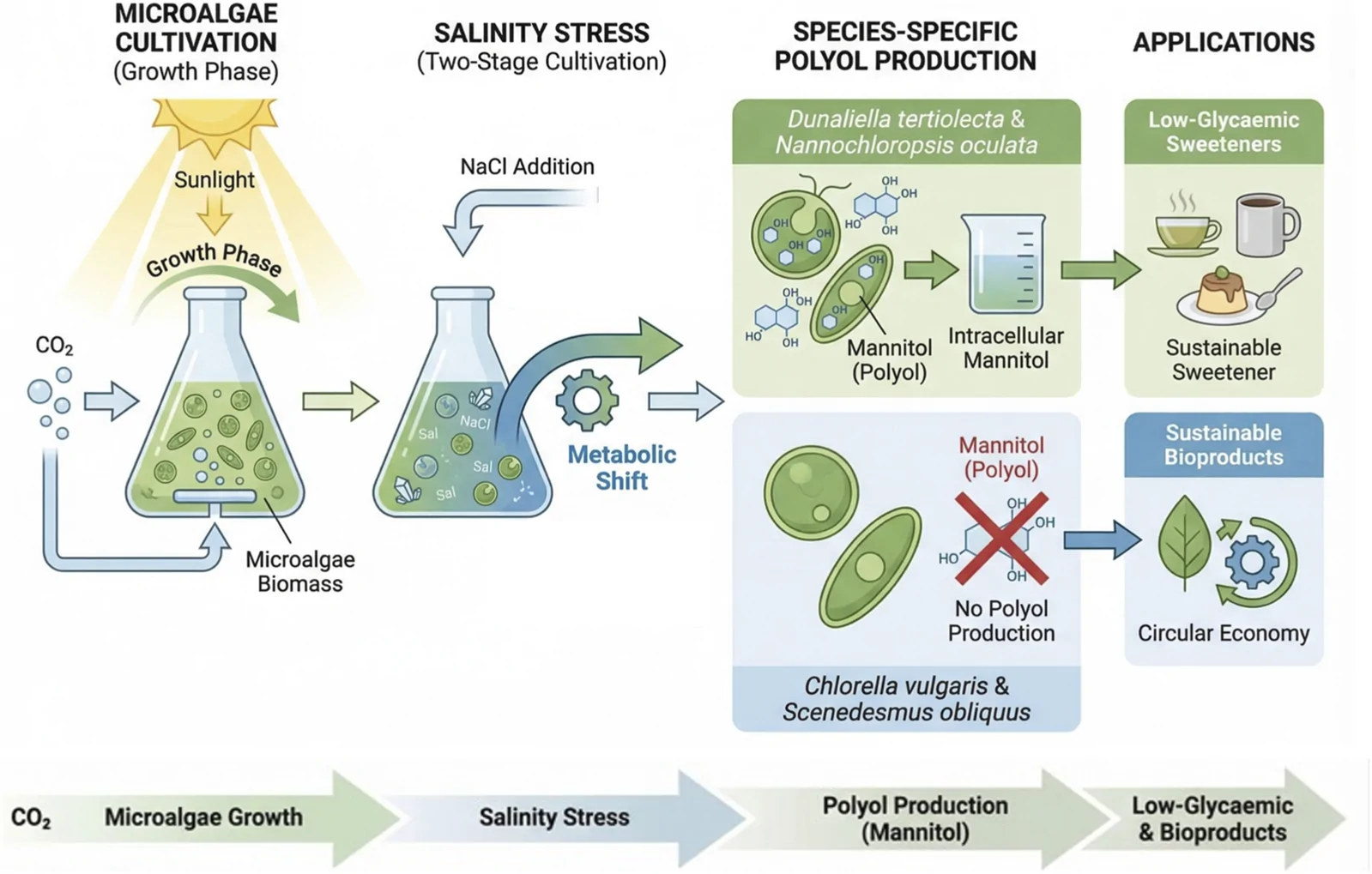

## 1 Introduction

The global prevalence of diabetes continues to rise, affecting more than 589 million adults aged 20–79 years and projected to exceed 853 million by 2050 (IDF Diabetes Atlas, 2025). The burden is particularly high in low- and middle-income countries, with approximately 43% of cases remaining undiagnosed, and global healthcare expenditures exceeding USD 1.015 trillion. These trends highlight the urgent need for safe and sustainable sugar alternatives (IDF Diabetes Atlas, 2025) Polyols, also known as sugar alcohols, including mannitol, sorbitol, and xylitol, are promising sugar substitutes, as they cause a significantly lower glycemic response (glycemic index, GI: 0–13) in contrast to conventional sugars such as sucrose (GI > 65) (Ghosh and Sudha, 2012). Currently, polyols are predominantly produced via chemical hydrogenation of sugars derived from starch or lignocellulosic feedstocks, processes that are energy-intensive and raise sustainability concerns due to land use and complex pretreatment requirements (Rice et al., 2020). First-generation crops, including sugarcane, corn, and sugar beet, are major sources of sugar; however, their use raises concerns about competition for arable land and food security. Conversely, lignocellulosic biomass, although abundant and non-edible, is highly recalcitrant and necessitates complex, multistage pretreatment and hydrolysis under severe physicochemical conditions to liberate sugars. These techniques are generally energy-intensive and environmentally harmful, impacting the overall sustainability of traditional sugar alcohol production methods (Liu et al., 2014; Mayers et al., 2018).

Microalgae have emerged as a viable and sustainable option for the production of polyols and other bioactive metabolites, owing to their unique physiological and ecological advantages. Several microalgal species synthesize polyols in response to salinity stress, making them attractive candidates for biological polyol production without the harsh processing conditions associated with conventional chemical routes (Carrasco-Reinado et al., 2019; Fajardo et al., 2020; Iwamoto and Shiraiwa, 2005). In addition, these microorganisms possess exceptional biochemical diversity and metabolic flexibility (Dolganyuk et al., 2020). They contain high-value bioactive compounds, including carbohydrates, proteins, and lipids, which have potential applications in the food, pharmaceutical, and biofuel industries (Borowitzka et al., 1977).

Polyol accumulation in microalgae is commonly induced by osmotic stress, particularly elevated salinity, where compounds such as mannitol function as compatible solutes that contribute to osmotic balance and stress tolerance. Studies on the red alga Caloglossa demonstrated rapid and reversible salinity-dependent regulation of intracellular mannitol, highlighting its role as an osmoprotectant (Karsten and West, 1993; Mostaert et al., 1995b; 1995a). Although environmental factors such as salinity, nutrient availability, and light regime can influence polyol accumulation (Borowitzka et al., 1977) previous studies have largely focused on physiological adaptation rather than evaluating microalgae as potential polyol-producing platforms.

Limited studies have reported polyol recovery from microalgal biomass. Carrasco-Reinado et al. (2019) demonstrated the extraction of sugars and polyols from several species, including Nannochloropsis gaditana, Arthrospira platensis (Spirulina), and Pyrocystis lunula, employing various extraction methods, such as mechanical and ultrasonic lysis, under different treatment conditions, and identified Nannochloropsis species as viable sources of mannitol. Margellou et al. (2024) further demonstrated the potential of microalgae for polyol production within an integrated biorefinery through downstream catalytic conversion. However, these studies focus primarily on post-harvest processing rather than stress-induced biosynthesis during cultivation. Together, these studies identify microalgae as an underexplored source of polyols but also reveal the need for comparative screening of stress-induced intracellular accumulation during cultivation.

Salinity stress is a major abiotic factor affecting microalgal metabolism. High salinity imposes osmotic and ionic stress and can promote the accumulation of compatible solutes, including mannitol, sorbitol, and glycerol, that contribute to cellular osmotic balance (Mostaert et al., 1995b). Previous studies on salinity stress in microalgae have primarily focused on growth performance and lipid accumulation for biofuel production (Chokshi et al., 2017; Pandit et al., 2017; Yun et al., 2019). Other studies have examined pigment modulation and carbohydrate accumulation under stress (Abidin et al., 2021; El-Katony and El-Adl, 2020; McLachlan, 1961; Papapanagiotou et al., 2024; Tammam et al., 2011). A detailed systematic screening of microalgal species for polyol production under salinity stress, particularly in the context of candidate selection for biological polyol production, remains limited and has not been addressed. A distinct knowledge gap exists in identifying microalgal species that could accumulate polyols under salinity stress and in characterizing the associated changes in biomass composition and nitrogen assimilation.

Nitrogen assimilation is closely associated with microalgal growth and biochemical composition (Geider and La Roche, 2002; Raven and Geider, 2003). Under optimal conditions, nitrogen uptake supports protein synthesis and biomass formation, whereas salinity or nutrient stress may reduce nitrogen assimilation and coincide with increased accumulation of nitrogen-poor compounds, including carbohydrates, lipids, pigments, and osmolytes (Breuer et al., 2015; Fernandes et al., 2016; Liang et al., 2023).

This study systematically screened two freshwater species, *Chlorella vulgaris* and *Scenedesmus obliquus*, and two marine species, *Dunaliella tertiolecta* and *Nannochloropsis oculata*, for intracellular polyol accumulation under salinity stress using a two-stage cultivation strategy. Changes in biomass composition (carbohydrates, proteins and pigments) and nitrogen assimilation were evaluated to identify species exhibiting favorable stress responses and to establish a framework for future optimization of microalgal polyol production. The conceptual framework is illustrated in Figure 1.

**FIGURE 1:**
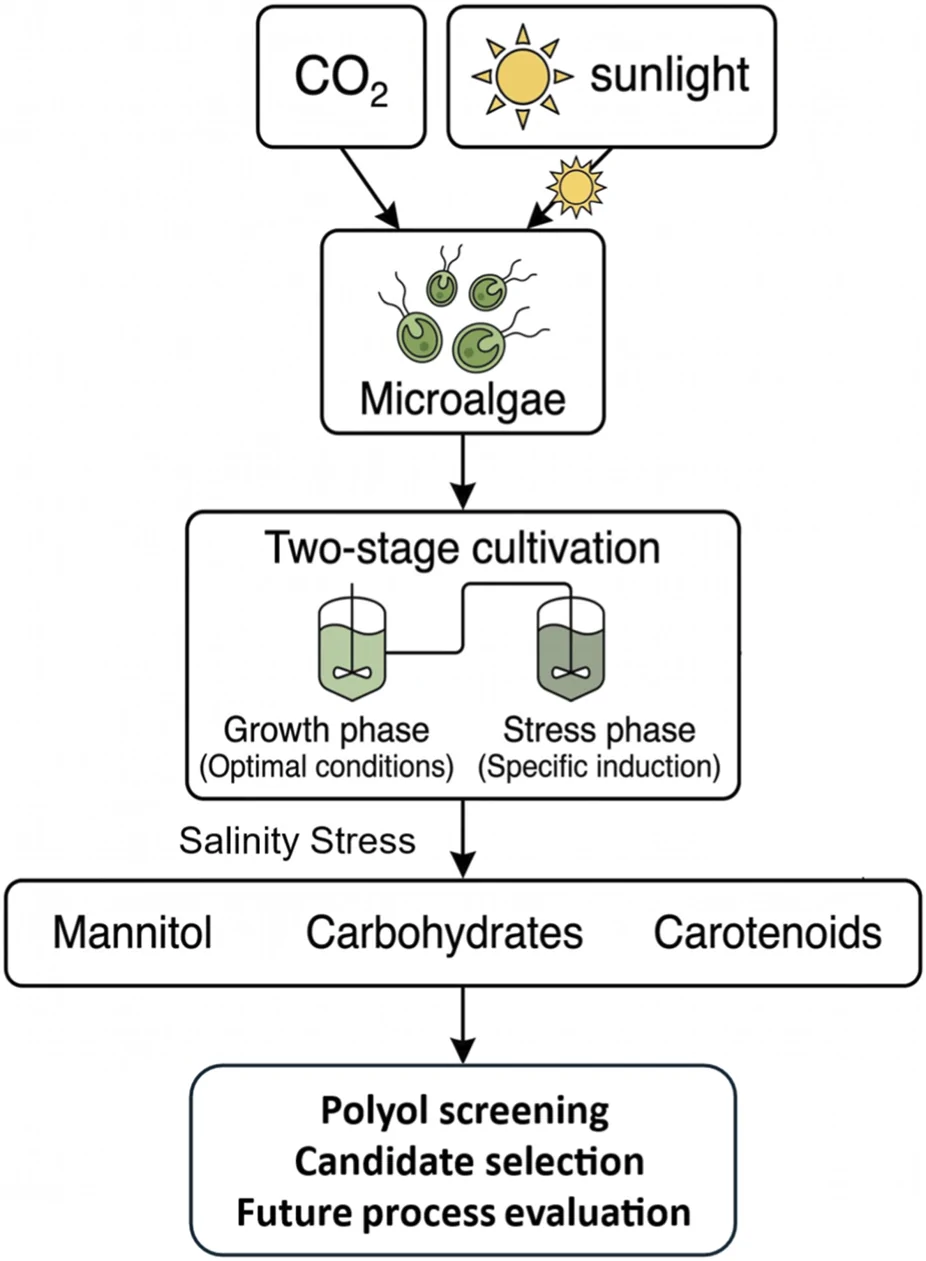
Conceptual framework linking salinity-induced metabolic responses with intracellular mannitol accumulation in microalgae. CO_2_ and sunlight are converted into microalgal biomass during an initial growth phase, followed by salinity stress induction in a two-stage cultivation process. The resulting stress response is associated with changes in mannitol, carbohydrates, and carotenoids, providing a basis for evaluating microalgae as potential sources of polyols and other stress-associated metabolites.

## 2 Materials and methods

### 2.1 Microalgae strains and cultivation conditions

Four microalgal strains were selected for this study, comprising two freshwater species, *Chlorella vulgaris* (CCAP 211/21A) and *Scenedesmus obliquus* (CCALA 454), and two marine species, *Dunaliella tertiolecta* (CCAP 19/25) and *Nannochloropsis oculata* (CCAP 849/7). All cultivation experiments were performed using two independent biological cultures (n = 2) for each treatment. Each culture bottle represented an independent experimental unit throughout the study. Marine species were cultivated in F/2 medium, while freshwater species were grown in BG11 medium. Cultures were maintained in 1 L Schott bottles (GL45, three-port caps) containing 1 L of medium, aerated continuously with filtered (0.22 µm, PTFE) ambient air at a rate of 500 mL min^-1^, controlled by a rotameter. No additional CO_2_ supplementation was provided.

Continuous illumination (24 h light:0 h dark) at a light intensity of approximately 2,500 lux (35 μmol m^-2^ s^-1^), and temperature was maintained at 25 °C ± 1 °C. Continuous illumination was used to maintain consistent laboratory cultivation conditions and support biomass production during the screening experiment. This approach has been widely adopted in laboratory-scale studies to enhance productivity and evaluate stress-induced metabolic responses under controlled conditions (Chokshi et al., 2017; Raven and Geider, 2003). Cultures were manually shaken twice daily and before sampling to ensure homogeneity, and these conditions were considered the base (non-stress) reference.

A two-stage cultivation strategy was employed to investigate the effects of salinity stress on polyol synthesis and biomass composition (Figure 2). In the pre-stress phase, cultures were inoculated into fresh medium under standard growth conditions, with initial optical densities (OD_750_ = 0.1) standardized to ensure equal starting biomass across treatments. Baseline OD, dry weight, and nitrate concentration were recorded on day 0, and cultures were grown for 6 days to reach exponential phase.

**FIGURE 2:**
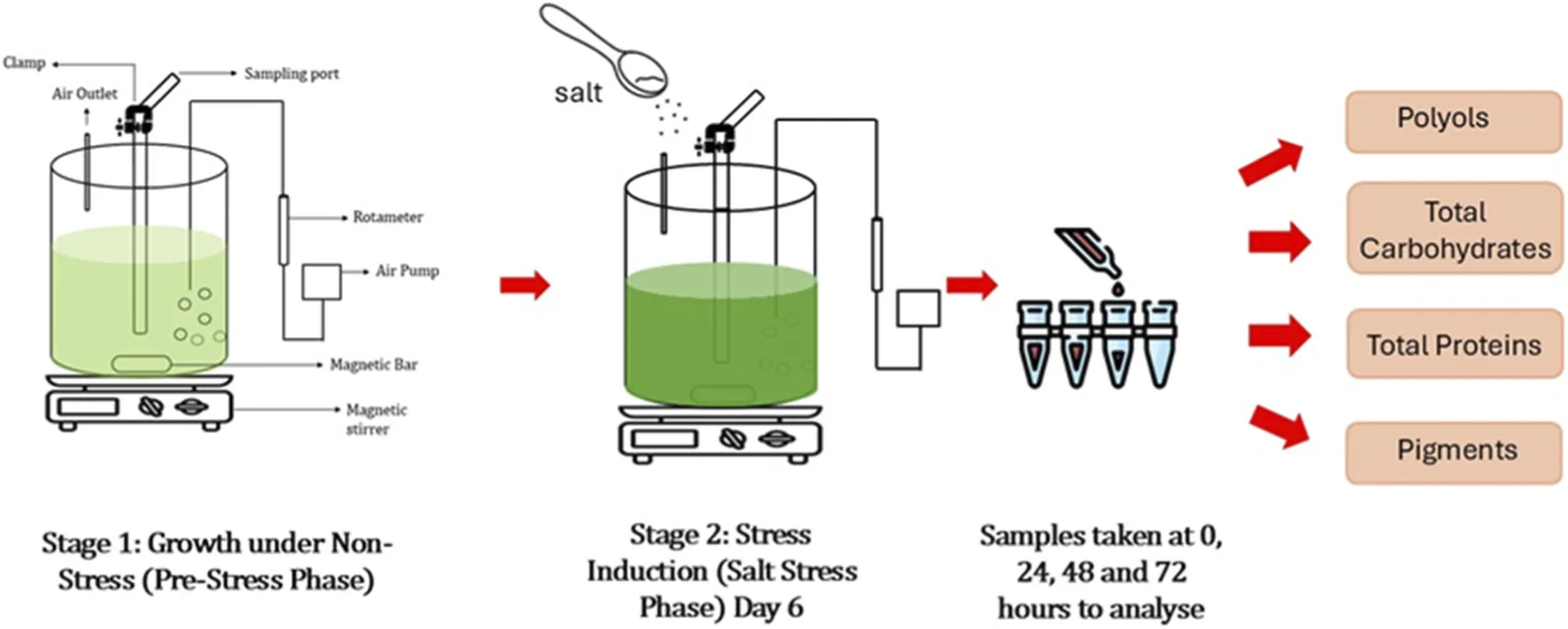
Two-stage cultivation strategy used for microalgal screening. Cultures were first grown under non-stress conditions and subsequently exposed to defined salinity treatments. Samples were collected at 0, 24, 48, and 72 h after stress induction for polyol, carbohydrate, protein, and pigment analyses.

On day 6, salinity stress was introduced by supplementing cultures with sea salt to final concentrations of 16 g L^-1^ and 32 g L^-1^ for freshwater strains, and 60 g L^-1^ and 80 g L^-1^ for marine strains. Salt addition was performed stepwise, with one-third of the total amount added at 2 h intervals to minimize osmotic shock. During the stress phase (days 6–9), OD_750_, dry weight, and nitrate concentration were monitored daily. Samples were also collected on days 6, 7, 8, and 9 for analyses of polyols, total carbohydrates, proteins, chlorophylls, and carotenoids.

### 2.2 Evaluation of growth and nitrate consumption

Optical density was measured at 750 nm using a spectrophotometer (Hach DR3900). Growth was monitored by measuring optical density at 750 nm using a Hach DR3900 spectrophotometer. This wavelength was selected to minimize interference from photosynthetic pigments during biomass estimation (Gaignard et al., 2019; Griffiths et al., 2011). To identify the appropriate wavelength, a spectral analysis was performed on high-density cultures of all four strains from 400 to 1,000 nm. Nitrate concentration in the culture medium was quantified as NO_3_–N (mg L^-1^) using a commercial cuvette test kit (Hach® LCK 339) following the manufacturer’s protocol. To minimize interference from chloride ions, which can affect colorimetric detection at high salinity, samples were appropriately diluted prior to analysis to maintain chloride concentrations below 5 g L^-1^. Dry weight was quantified according to standard methods at 105 °C. Briefly, a specified volume (10 mL) of culture was filtered using pre-weighed Whatman GF/F filters. The biomass retained on the filter was washed twice with deionized water to remove residual salts before drying. The filters were subsequently dried at 105 °C until a constant weight was attained, cooled in a desiccator, and reweighed. The dry weight was determined by subtracting the original filter weight from the final filter weight.

### 2.3 Analysis of polyols using HPLC

A 10 mL aliquot of microalgal culture was harvested by centrifugation at 4,700 rpm for 10 min at 20 °C. The supernatant was discarded, and the harvested cell pellet was washed once with deionized water before extraction. The resulting pellet was resuspended in 1 mL of deionized (DI) water. Cell disruption was carried out using probe ultrasonication at 0 °C for 10 min in the pulsed mode (3 s ON, 1 s OFF) at 35% amplitude. Following ultrasonication, the lysate was centrifuged again at 4,700 rpm for 10 min at 20 °C. The resulting supernatant was filtered through a 0.22 µm syringe filter and subjected to HPLC analysis.

Polyol analysis was performed using a Waters Alliance HPLC system equipped with an Atlantis Premier BEH Z-HILIC column (4.6 × 150 mm, 2.5 µm; p/n: 186009994). The mobile phase consisted of deionized water and acetonitrile. Detection was performed using an evaporative light-scattering detector (ELSD). The flow rate was 0.4 mL min^-1^, and the injection volume was 5 µL. Quantification was performed using external calibration with mixed polyol standards (0.05–2.00 mg mL^-1^) including glycerol, arabitol, xylitol, sorbitol, mannitol, and inositol. Calibration curves were fitted using quadratic regression because of the nonlinear response of the ELSD detector. The coefficients of determination (R^2^) ranged from 0.991 to 0.995 for the individual polyols, and the method detection limit was 0.05 mg mL^-1^. Detailed chromatographic conditions, ELSD operating parameters, and the gradient program are provided in Supplementary Tables S4–S6 and Supplementary Figure S1.

### 2.4 Total carbohydrates analysis

The total carbohydrate content was determined using the phenol–sulfuric acid method in a microplate format, following the protocol described by Ogura et al. (2023). Sample preparation was identical to that used for HPLC analysis. Briefly, 30 μL of the prepared sample was transferred to each well of a flat-bottomed 96-well microplate. Subsequently, 150 μL of concentrated sulfuric acid (H_2_SO_4_) was added to each well, and the plate was incubated at 90 °C for 15 min. After cooling to room temperature, 30 μL of a 5% phenol solution was added to each well, followed by incubation at room temperature for 60 min. Absorbance was measured at 490 nm using a microplate reader. Carbohydrate concentrations were calculated from a glucose calibration curve.

### 2.5 Total protein analysis

The total protein content was quantified using a bicinchoninic acid (BCA) protein assay kit (Thermo Fisher Scientific, United States), following the manufacturer’s protocol. Sample preparation was identical to that used for HPLC analysis. For each assay, 50 μL of the sample was transferred into the wells of a 96-well microplate, followed by the addition of 100 μL of BCA working reagent. The contents were thoroughly mixed and incubated at 50 °C for 30 min. After cooling to room temperature, absorbance was measured at 562 nm using a microplate reader. Protein concentration was determined from a bovine serum albumin (BSA) calibration curve.

### 2.6 Pigment analysis

Pigment extraction was performed using 90% (v/v) acetone, as described by Reshma et al. (2021). In brief, a 10 mL aliquot of microalgal culture was harvested by centrifugation at 4,700 rpm for 10 min at 20 °C. The supernatant was discarded, and the resulting pellet was resuspended in 10 mL of 90% acetone. Samples were homogenized by probe ultrasonication at 0 °C for 5 min using a pulsed cycle of 3 s ON and 1 s OFF at 35% amplitude and centrifuged again at 4,700 rpm for 10 min, and the absorbance of the clear supernatant was measured at 663, 645, and 470 nm using a UV-visible spectrophotometer (Hach DR 3900).

Chlorophyll a, chlorophyll b, total chlorophyll, and total carotenoids were calculated using Equations 1–4, based on absorbance values at the respective wavelengths (Arnon, 1949; Lichtenthaler, 1987). Pigment contents were normalized to biomass dry weight and expressed as mg g^-1^ DW.

$$\text{Chlorophyll }a\;\left(\mu {g\;\text{mL}}^{-1}\right)=12.7A_{663}-2.69A_{645}$$ (1)

$$\text{Chlorophyll }b\;\left(\mu {g\;\text{mL}}^{-1}\right)=22.4A_{645}-4.68A_{663}$$ (2)

$$\text{Total chlorophyll }\left(\mu {g\;\text{mL}}^{-1}\right)=8.02A_{663}+20.2A_{645}$$ (3)

$$\text{Carotenoids }\left(\mu {g\;\text{mL}}^{-1}\right)=\frac{1000A_{470}-1.63\left(\text{Chlorophyll}\;a\right)-104.9\left(\text{Chlorophyll}\;b\right)}{221}$$ (4)

### 2.7 Statistical analysis

Statistical analyses were performed using Minitab® Statistical Software (Minitab LLC, United States). The experimental unit for all statistical analyses was the independent biological culture (n = 2 per treatment). Carbohydrates, proteins, and pigments were quantified in analytical triplicate for each biological culture, and the analytical replicates were averaged prior to statistical analysis. Polyol measurements were obtained from a single HPLC analysis of each biological culture. Two-way analysis of variance (ANOVA) was used to evaluate the effects of salinity treatment and stress duration on the measured parameters. Polyol concentrations were not detected in control cultures for several species; therefore, statistical analysis of polyol accumulation was restricted to salinity-treated cultures. Model assumptions were evaluated qualitatively using residual plots. Owing to the limited number of biological replicates, statistical analyses were used to support observed biological trends and were interpreted together with the consistency of responses across independent cultures.

## 3 Results and discussion

One of the main issues in producing microalgae-based bioproducts under stressed conditions is the conflict regarding the optimum conditions required for a high rate of growth and a high bioproduct yield (Park et al., 2019). Microalgae grown under constant stress may induce stress-associated metabolite accumulation; however, their growth rate is also reduced, which lowers their total productivity and ultimately increases the cost of the bioproducts (Chokshi et al., 2017). Two-stage cultivation separates biomass growth from stress induction and can therefore reduce the conflict between biomass accumulation and metabolite formation under continuous stress. In this study, cultures were first grown under non-stress conditions and subsequently exposed to salinity to evaluate species-specific intracellular polyol accumulation and changes in biomass composition. This strategy has been proposed in several studies on the microalgal production of biofuels (Rashid et al., 2015; Widjaja et al., 2009; Yun et al., 2019), carbohydrates (Chong et al., 2019; Wang et al., 2015), and other high-value products (Liyanaarachchi et al., 2021).

### 3.1 Salinity-induced variations in growth, nitrogen uptake, and macromolecular distribution in *Chlorella vulgaris*

Minor differences in growth and nitrate uptake were observed in *Chlorella vulgaris* cultures subjected to salinity stress (16 and 32 g L^-1^) in comparison to the control (Figure 3a). Although slight variations in optical density were observed among treatments, biomass growth remained comparable throughout the experiment (Figure 3a). Statistical analysis did not identify a significant effect of salinity on growth (p > 0.05), indicating that the tested salinity levels did not substantially impair biomass accumulation. Nitrate uptake closely correlated with biomass growth, indicating continued nitrate uptake during cultivation in BG11 medium.

**FIGURE 3:**
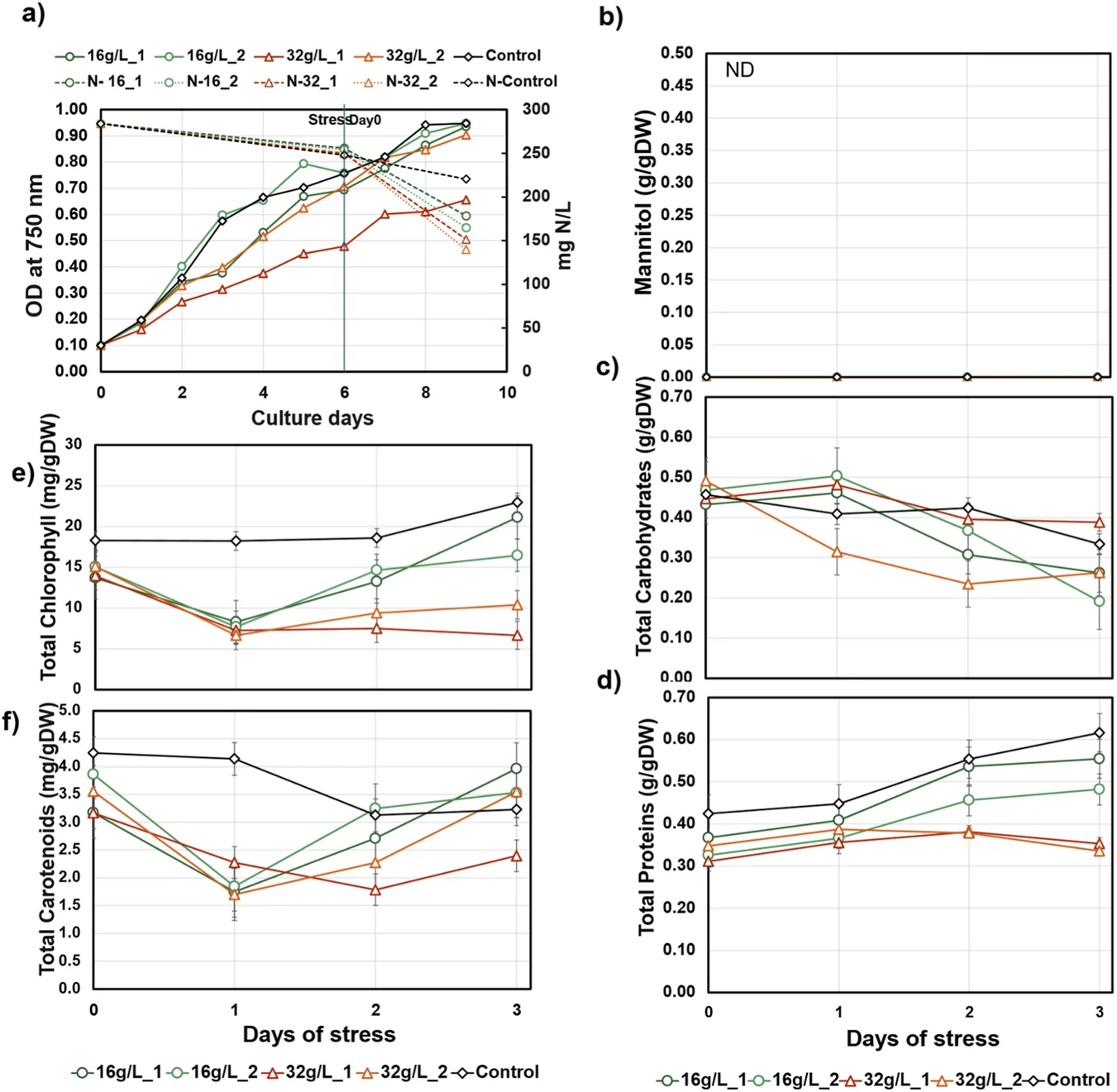
Effects of salinity stress on *Chlorella vulgaris*: **(a)** growth and nitrate concentration; **(b)** intracellular mannitol; **(c)** total carbohydrates; **(d)** total proteins; **(e)** total chlorophyll; and **(f)** total carotenoids. Salinity treatments of 16 and 32 g L^-1^ were initiated on cultivation day 6, designated stress day 0. Unless otherwise stated, all biochemical measurements represent the mean of two independent biological cultures (n = 2), with analytical triplicates averaged prior to statistical analysis.

None of the analyzed polyols (mannitol, sorbitol, xylitol, arabitol, glycerol, or inositol) were detected in *C. vulgaris* under either control or salinity-stressed conditions, at any sampling interval (Figure 3b), showing that *C. vulgaris* did not contain detectable levels of polyols under the applied salinity conditions. The absence of detectable polyols in freshwater species was confirmed relative to the method detection limit of the HPLC–ELSD system (limit of detection 0.05 mg mL^-1^), indicating that polyol concentrations, if present, were below quantifiable levels. Carbohydrate content responded to salinity stress (Figure 3c). Under moderate salinity (16 g L^-1^), carbohydrate levels increased transiently during the initial stress period before declining, whereas high salinity (32 g L^-1^) resulted in a sustained decrease from approximately 44% DW to below 35% DW. Statistical analysis supported a significant effect of salinity treatment (p = 0.016), whereas stress duration had no significant influence (p = 0.875). The control cultures had a relatively stable carbohydrates content.

The protein content showed considerable variation over time and in response to treatment (Figure 3d). Protein levels rose about 20% in both control and 16 g L^-1^ cultures over the 3 days, but cultures subjected to 32 g L^-1^ experienced a decrease of approximately 7%, which was associated with the high-salinity treatment. Statistical analysis supported significant effects of both salinity (p < 0.001) and stress duration (p = 0.007).

Salinity stress significantly affected photosynthetic pigments. Chlorophyll content declined by approximately 42% under 32 g L^-1^ salinity, whereas partial recovery was observed under 16 g L^-1^ salinity (Figure 3e). This response is in agreement with salinity-induced impairment of the photosynthetic apparatus reported in previous studies (Yun et al., 2019), although photosynthetic performance was not directly assessed in the present study. Statistical analysis confirmed significant effects of both salinity treatment (p < 0.001) and stress duration (p = 0.018). The carotenoid concentration exhibited substantial variation over time (p = 0.004), whereas the primary effect of treatment was not statistically significant (p = 0.115) (Figure 3f). Carotenoid content was more stable compared to chlorophyll under salinity stress. These trends highlight that *C. vulgaris* retained carotenoid content more effectively than chlorophyll under moderate stress, whereas high salinity (32 g L^-1^) severely compromises pigment stability. Carotenoids play an important photoprotective role during stress. Carotenoid depletion was less pronounced than chlorophyll under salinity stress, suggesting a greater resilience of protective pigments during stress exposure. Previous studies have reported increased carotenoid accumulation in *C. vulgaris* under mild salinity (5–10 g L^-1^) combined with elevated light intensity (Ali et al., 2021). In contrast, the higher salinity levels applied in the present study resulted in a decline in carotenoid content, indicating that these conditions may have exceeded the tolerance threshold for enhanced carotenoid biosynthesis. Although oxidative stress markers were not measured, the observed pigment responses are consistent with previous reports describing salinity-induced impairment of the photosynthetic apparatus under severe stress. Notably, carotenoid depletion remained less pronounced than chlorophyll loss, suggesting that carotenoids retained a protective role despite reduced pigment stability.

### 3.2 Salinity-induced changes in growth, nitrate uptake, and biomass composition in *Scenedesmus obliquus*

*Scenedesmus obliquus* exhibited similar growth across all treatments during the pre-stress phase, followed by distinct salinity-dependent responses after stress induction (Figure 4a). Moderate salinity (16 g L^-1^) promoted biomass accumulation relative to the control, whereas high salinity (32 g L^-1^) gradually reduced growth. Nitrate uptake closely followed biomass accumulation throughout the experiment, indicating that nitrogen assimilation remained active under both salinity conditions.

**FIGURE 4:**
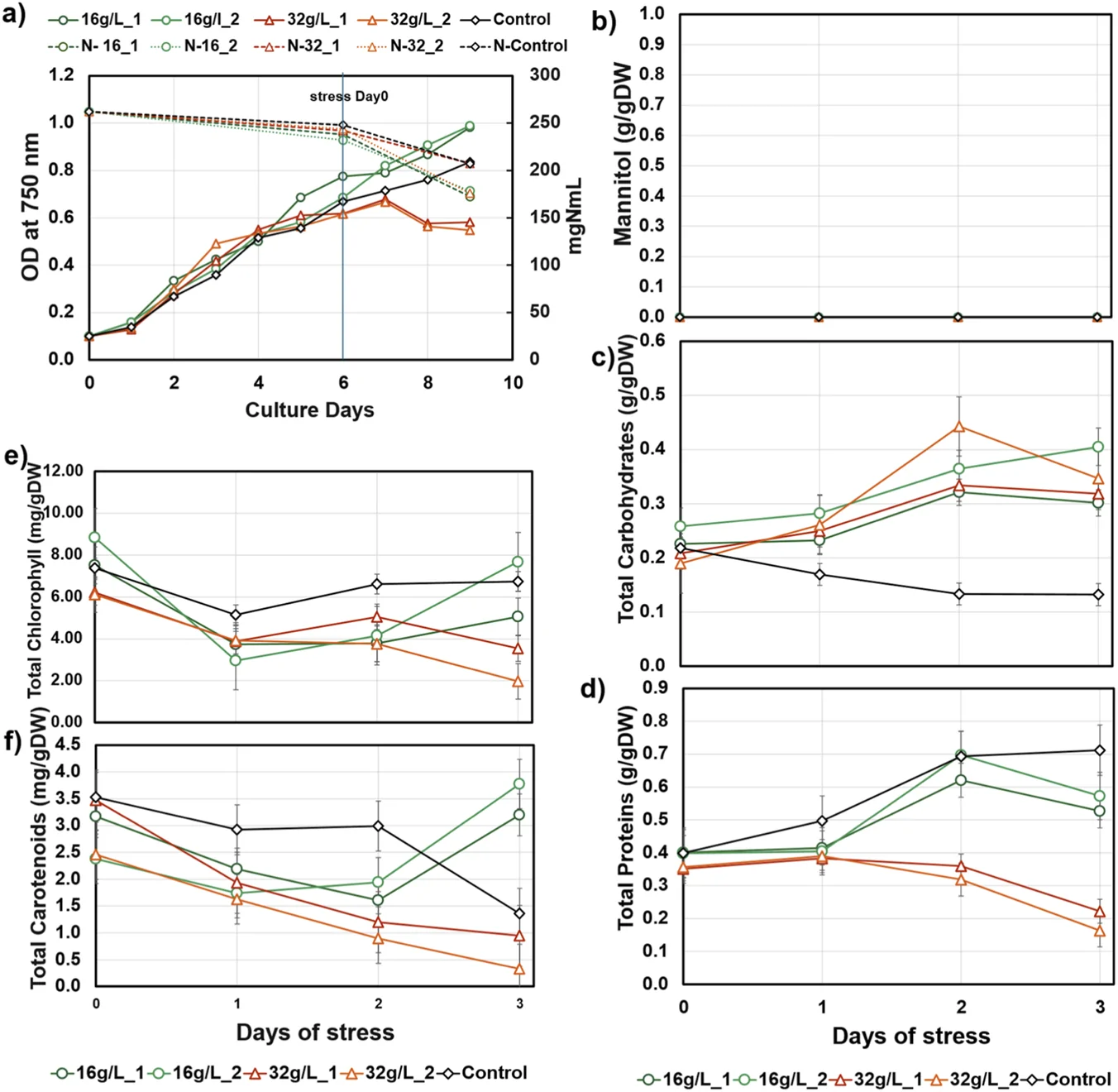
Effect of salinity stress on *Scenedesmus obliquus*: **(a)** growth and nitrate concentration; **(b)** intracellular mannitol; **(c)** total carbohydrates; **(d)** total proteins; **(e)** total chlorophyll; and **(f)** total carotenoids. Salinity treatments of 16 and 32 g L^-1^ were initiated on cultivation day 6, designated stress day 0.

HPLC analysis did not detect any of the polyols analyzed in either the control or salinity-treated cultures at any sampling point (Figure 4b). In contrast, carbohydrate content responded to salinity stress (Figure 4c). While carbohydrate levels remained relatively constant in the control cultures, both salinity treatments resulted in increases of approximately 10%–15% DW during the stress period. Statistical analysis supported significant effects of both salinity treatment (p = 0.004) and stress duration (p = 0.048).

Protein content (Figure 4d) increased by approximately 40% in the control by day 3 and under 16 g L^-1^ salinity by day 2. In contrast, the two cultures exposed to 32 g L^-1^ showed declines of approximately 37%–50% during the stress period. These reductions are consistent with salinity-associated changes in protein content previously reported for this species and related chlorophytes by Pancha et al. (2015). Nevertheless, as oxidative stress markers and protease activities were not measured, mechanistic interpretations are restricted to consistency with earlier studies rather than direct evidence.

Chlorophyll content (Figure 4e) declined by more than 50% within the first 24 h under both salinity treatments, indicating a rapid impairment of the photosynthetic apparatus. In contrast, carotenoids were comparatively better retained, particularly under 16 g L^-1^ salinity (Figure 4f), suggesting greater stability of photoprotective pigments during moderate stress. Similar responses have been reported in Chlamydomonas reinhardtii, where mild salinity promoted carotenoid accumulation while chlorophyll declined (Fal et al., 2022).

The observed growth stimulation under moderate salinity suggests that *S. obliquus* tolerated the applied osmotic stress under the conditions investigated. Continued nitrate uptake throughout the stress period indicates sustained nitrogen assimilation during salinity exposure, as previously reported for growth-phase-dependent salinity tolerance in *S. obliquus* (El-Katony and El-Adl, 2020). The lack of detectable polyols suggests that *S. obliquus* does not utilize polyol-based osmoprotection under the examined conditions. Rather, stress adaptation seems to entail changes in carbohydrate, protein, and pigment composition. This response differed from that observed in the marine species.

### 3.3 Salinity-induced changes in growth, nitrate uptake, and biomass composition in *Dunaliella tertiolecta*

*Dunaliella tertiolecta* exhibited consistent growth across all salinity conditions, demonstrating its halotolerant characteristics (Figure 5a). Biomass accumulation at salinities of 60 and 80 g L^-1^ showed similar trends between the two biological cultures at both salinity levels. Nitrate uptake closely followed biomass accumulation, indicating active nitrogen assimilation throughout cultivation.

**FIGURE 5:**
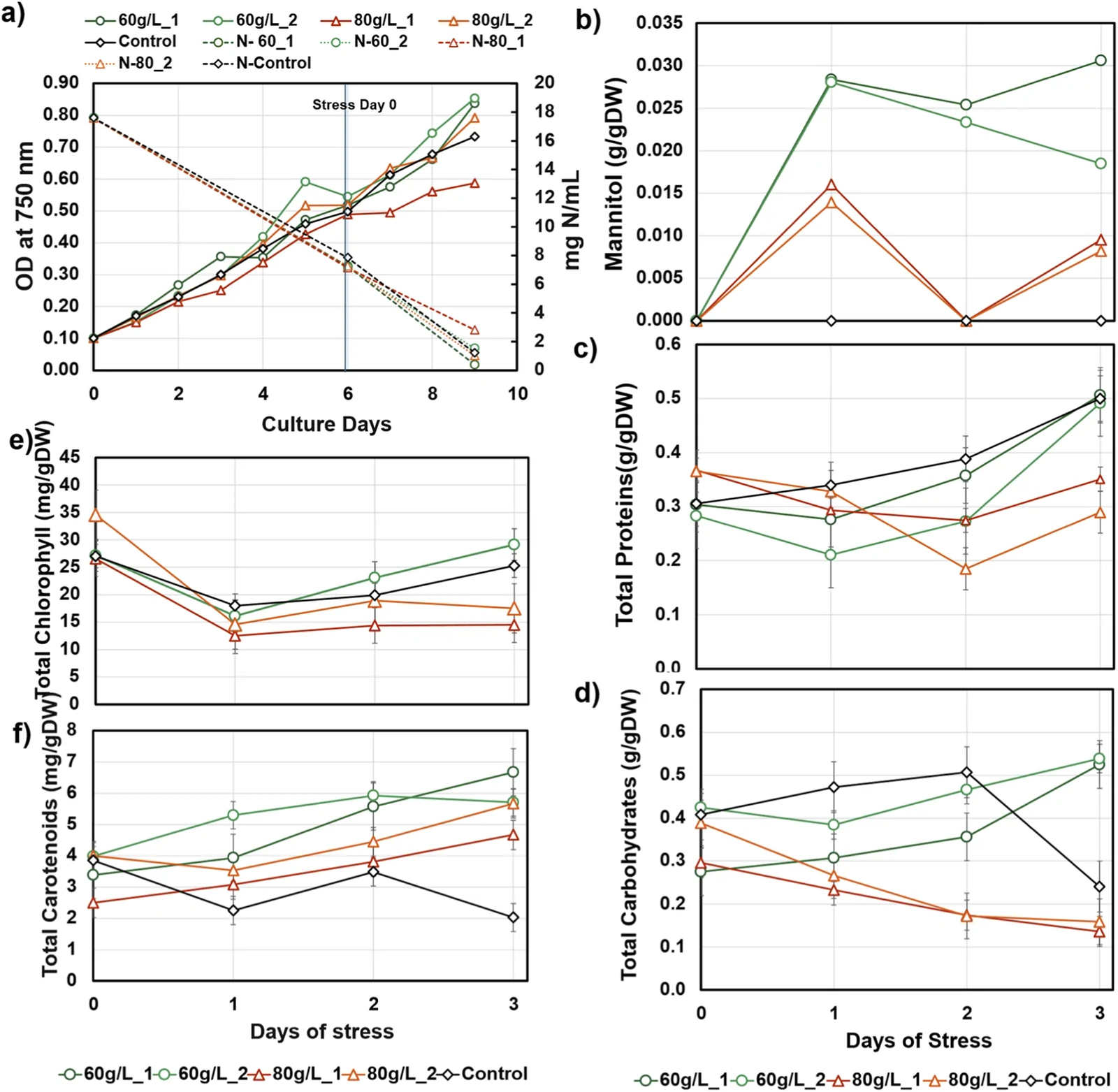
Effect of salinity stress on *Dunaliella tertiolecta*: **(a)** growth and nitrate concentration; **(b)** intracellular mannitol; **(c)** total carbohydrates; **(d)** total proteins; **(e)** total chlorophyll; and **(f)** total carotenoids. Salinity treatments of 60 and 80 g L^-1^ were initiated on cultivation day 6, designated stress day 0.

Among the polyols analyzed, mannitol was predominant, whereas inositol was detected at a lower concentration, reaching approximately 0.7% DW on stress day 3 (Figure 5b). Both polyols were detected only in salinity-treated cultures. Under moderate salinity (60 g L^-1^), intracellular mannitol reached a maximum of 28 mg g^-1^ DW (2.8% DW) after 24 h before gradually declining, a pattern that may reflect the transient accumulation of compatible solutes during the early stages of osmotic stress (Iwamoto and Shiraiwa, 2005). Although the metabolic fate of intracellular mannitol was not investigated in the present study, evidence from algae and other mannitol-producing photosynthetic organisms suggests that mannitol functions not only as a compatible solute but also as a dynamic carbon reserve that can be remobilized under prolonged stress to support cellular maintenance and energy metabolism (Iwamoto and Shiraiwa, 2005; Stoop et al., 1996). In these organisms, intracellular mannitol concentration reflects the balance between biosynthesis and subsequent utilization rather than continuous accumulation. Under severe salinity, increasing energetic demands associated with ion homeostasis, membrane repair, and antioxidant defence may therefore compete with continued mannitol accumulation (Iwamoto and Shiraiwa, 2005; Tonon et al., 2017), contributing to the transient patterns observed in *D. tertiolecta* and *N. oculata*. These interpretations remain speculative because metabolic fluxes and enzyme activities were not measured in the present study. In contrast, cultures exposed to 80 g L^-1^ accumulated substantially lower amounts, approaching the detection limit by the second day of stress. Statistical analysis supported significant effects of both salinity and stress duration on mannitol accumulation (p < 0.05).

Mannitol accumulation was accompanied by changes in biomass composition. Moderate salinity promoted carbohydrate accumulation to approximately 50% DW, whereas high salinity significantly reduced carbohydrate content to below 15% DW (p = 0.003) (Figure 5c). Protein content increased progressively in the control cultures from 30.6% DW to 49% DW over the stress period, while cultures exposed to 60 g L^-1^ showed a modest initial decline (4% DW) followed by recovery to levels comparable with the control (Figure 5d). This pattern is consistent with previous reports describing transient changes in protein content during salinity stress adaptation in *Dunaliella* (Tammam et al., 2011). Photosynthetic pigments also responded to salinity stress. Chlorophyll content declined approximately 1.7-fold within the first 24 h of stress before partially recovering under moderate salinity (Figure 5e). In contrast, carotenoid content increased by approximately 1.8-fold and 1.6-fold under 60 and 80 g L^-1^ salinity, respectively (Figure 5f). Similar increases in carotenoids have been reported in *Dunaliella* and similar taxa under salinity stress and are generally associated with enhanced photoprotection during environmental stress (Papapanagiotou et al., 2024; Tammam et al., 2011).

The present study focused exclusively on intracellular polyols and therefore did not quantify extracellular glycerol, which is recognized as the primary osmolyte in *Dunaliella* (Borowitzka et al., 1977; Oren, 2017). Consequently, the relative contribution of glycerol and mannitol to osmotic regulation cannot be determined from the current dataset. Nevertheless, the reproducible accumulation of intracellular mannitol under moderate salinity demonstrates that *D. tertiolecta* responds to salinity stress through detectable polyol accumulation, distinguishing it from the freshwater species examined in this study.

From a bioprocess perspective, the simultaneous accumulation of mannitol, carbohydrates, and carotenoids under moderate salinity highlights the potential of *D. tertiolecta* as a suitable candidate for further investigations of stress-induced polyol accumulation. Future studies should quantify both intracellular and extracellular osmolyte pools and evaluate cultivation strategies that improve volumetric mannitol productivity.

### 3.4 Salinity stress response in growth, nitrogen uptake, and metabolites in *Nannochloropsis oculata*

*Nannochloropsis oculata* exhibited high tolerance to salinity stress, maintaining approximately 65%–70% of the control biomass under elevated salinity throughout the stress period (Figure 6a). Despite reduced growth at higher salinity, nitrate uptake continued throughout the experiment, indicating sustained metabolic activity.

**FIGURE 6:**
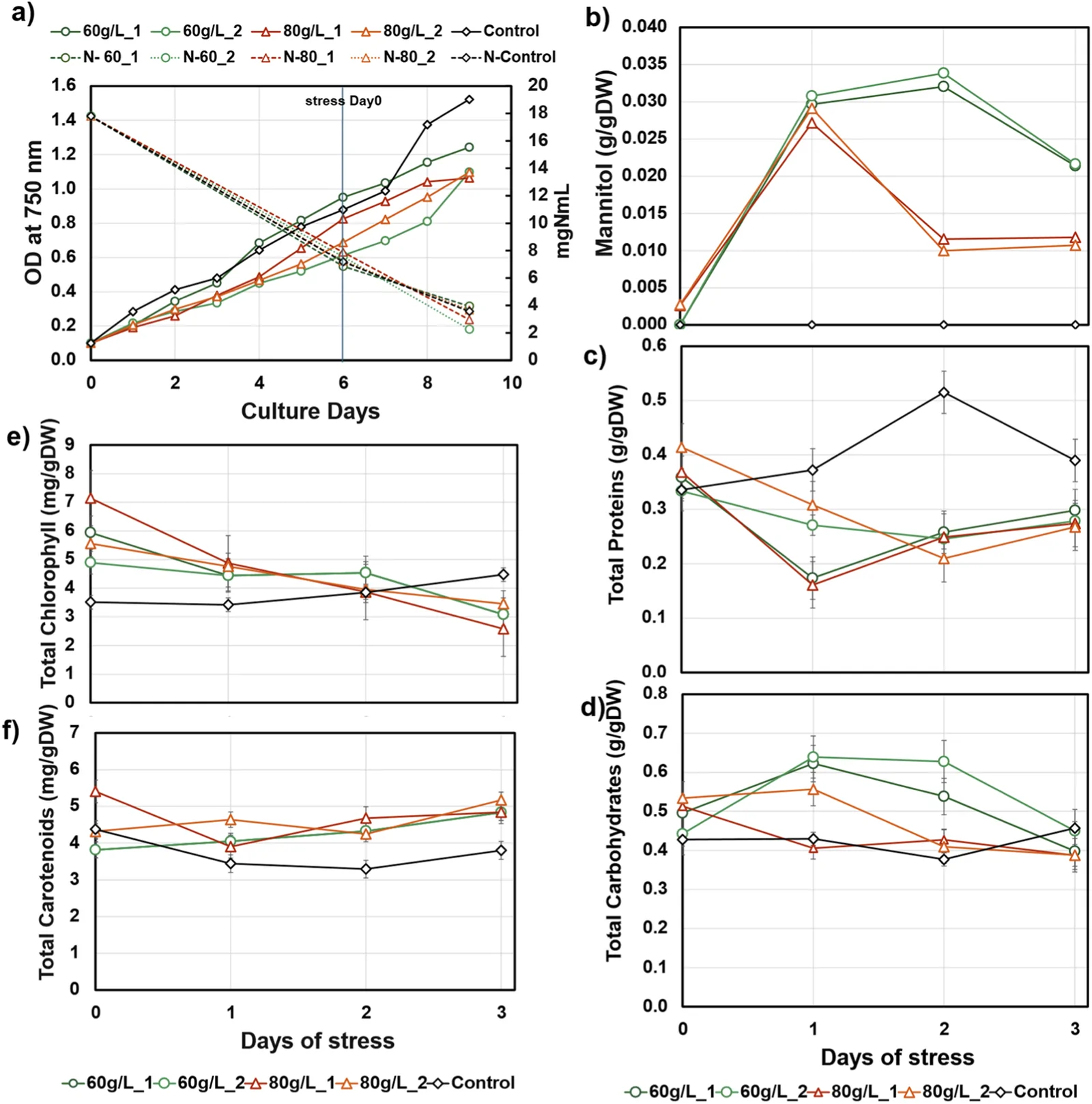
Effect of salinity stress on *Nannochloropsis oculata*: **(a)** growth and nitrate concentration; **(b)** intracellular mannitol; **(c)** total carbohydrates; **(d)** total proteins; **(e)** total chlorophyll; and **(f)** total carotenoids. Salinity treatments of 60 and 80 g L^-1^ were initiated on cultivation day 6, designated stress day 0.

Figure 6b illustrates the induction of intracellular mannitol accumulation following salinity stress. Mannitol was the predominant intracellular polyol detected in *N. oculata*, while low concentrations of inositol (0.9% DW on Day 2) and sorbitol (0.7% DW on Day 3) were also detected. Under moderate salinity (60 g L^-1^), mannitol reached a maximum of 34 mg g^-1^ DW after 2 days of stress, whereas lower concentrations were observed at 80 g L^-1^ salinity. Statistical analysis supported significant effects of both salinity and stress duration on mannitol accumulation (p = 0.002). Mannitol levels subsequently declined after day 2, suggesting a transient response to osmotic stress. A similar transient pattern was observed in *D. tertiolecta*, suggesting that intracellular mannitol accumulation represents an early osmotic response rather than a metabolite that increases continuously with salinity or stress duration. The possible metabolic basis for this behaviour is discussed in Section 3.3. Because polyols were below the detection limit in the control cultures, statistical analysis was restricted to the salinity-treated samples. The accumulation of mannitol is consistent with a role in osmotic adjustment in *N. oculata*. The accumulation of intracellular mannitol under salinity stress is consistent with its reported role as an osmoprotective metabolite in algae (Iwamoto and Shiraiwa, 2005). The lack of detectable polyols in control cultures indicates that detectable mannitol accumulation was associated with salinity exposure under the tested conditions.

Carbohydrate content increased under moderate salinity, reaching approximately 1.5-fold that of the control after 24–48 h of stress (Figure 6c). Although the overall treatment effect was modest (p = 0.054), carbohydrate accumulation under 60 g L^-1^ was significantly higher than the control (p = 0.017), whereas no significant change was observed under 80 g L^-1^. The protein content decreased during salt stress, with the largest observed at 80 g L^-1^ salinity (32.4% decrease from day 0 to day 3 of stress) (Figure 6d). This decrease was particularly evident during the initial stress phase and did not completely recover by day 3, indicating a persistent impairment in protein accumulation under elevated salinity stress. The decrease in protein content is consistent with a salinity associated changes in nitrogen containing biomass components towards stress adaptation, as previously reported in Nannochloropsis species (Khatoon et al., 2014). The total chlorophyll content decreased under both 60 g L^-1^ (17%) and 80 g L^-1^ (24%) salinity during the initial 24 h of stress, subsequently exhibiting partial stability (Figure 6e). In contrast, overall carotenoid content increased 1.2-fold under salinity stress, with elevated levels noted in both 60 g L^-1^ and 80 g L^-1^ salinity treatments relative to the control (Figure 6f), indicating preferential maintenance of photoprotective pigments. Similar responses have been reported in salinity-stressed Nannochloropsis species (Gu et al., 2012).

*Nannochloropsis oculata* maintained substantial growth under elevated salinity, confirming its inherent halotolerance, a characteristic previously reported for marine eustigmatophytes (Gu et al., 2012). The lower mannitol accumulation observed at 80 g L^-1^ compared with 60 g L^-1^ suggests that severe salinity may increase cellular maintenance demands, thereby limiting continued polyol accumulation. Additionally, continued biomass accumulation during the stress period may have contributed to a reduction in biomass-specific mannitol concentrations through dilution of intracellular polyol pools. Moderate salinity (60 g L^-1^) promoted intracellular mannitol and carbohydrate accumulation, whereas higher salinity (80 g L^-1^) was associated with lower protein content and increased carotenoid accumulation. These responses suggest that different salinity levels influence biomass composition in distinct ways. Although statistical significance was not observed at every individual sampling point, consistent trends were evident throughout the stress period, indicating reproducible physiological responses despite the limited biological replication. Overall, *N. oculata* exhibited the highest intracellular mannitol accumulation among the four species evaluated, together with coordinated changes in carbohydrates, proteins, and pigments, identifying it as a suitable candidate for future studies aimed at optimizing stress-induced polyol accumulation.

### 3.5 A comparative overview of salinity-induced metabolic responses

The comparative screening revealed clear species-specific responses to salinity stress (Figure 7). Intracellular mannitol accumulation was detected exclusively in the two marine species, *D. tertiolecta* and *N. oculata*, whereas neither freshwater species accumulated detectable polyols under the tested conditions. In contrast, *C. vulgaris* and *S. obliquus* responded to salinity through changes in carbohydrate, protein, and pigment composition, indicating alternative stress adaptation strategies. These findings demonstrate that intracellular mannitol accumulation is not a universal response to salinity but is strongly species dependent.

**FIGURE 7:**
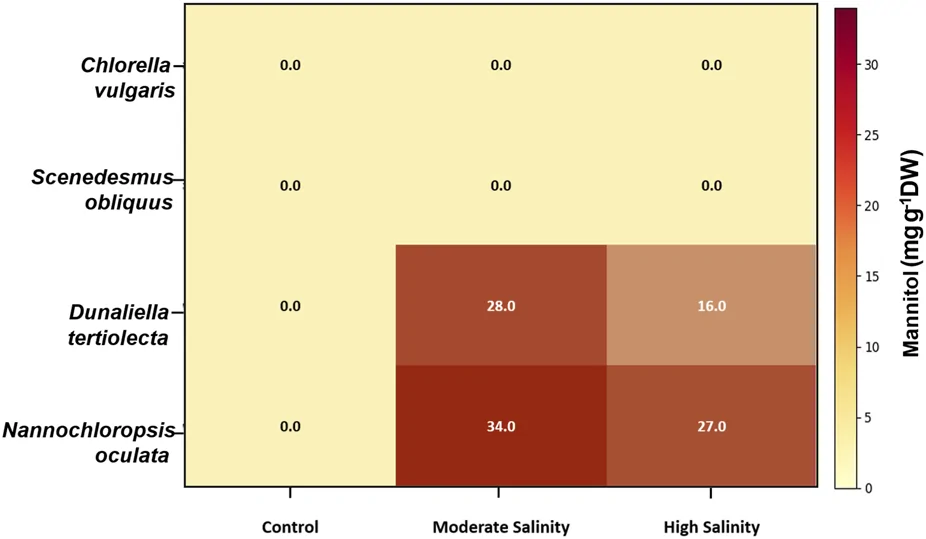
Heat map of salinity-induced mannitol accumulation across four microalgal species. Relative mannitol levels (mg g^-1^ DW) measured during the stress phase are shown for four species under moderate and high salinity along with control. Color intensity indicates polyol abundance. Polyols were detected exclusively in *Dunaliella tertiolecta* and *Nannochloropsis oculata*, revealing distinct species-specific osmoadaptive strategies.

To facilitate comparison on both a biomass and culture basis, maximum intracellular mannitol accumulation was expressed as mg g^-1^ DW and converted to volumetric concentration (mg L^-1^ culture) (Table 1). Volumetric concentration was calculated from maximum intracellular mannitol accumulation and corresponding biomass concentration at the same sampling point. *Nannochloropsis oculata* exhibited the highest intracellular mannitol accumulation at 60 g L^-1^ salinity (34 mg g^-1^ DW), corresponding to 17.68 mg L^-1^ culture. Although mannitol accumulation decreased under 80 g L^-1^ salinity, volumetric concentrations remained comparable due to sustained biomass levels. At 80 g L^-1^, volumetric mannitol concentration remained close to the moderate-salinity value in *N. oculata* but decreased substantially in *D. tertiolecta*. A comparison between the scope of the present study and representative literature is provided in Supplementary Table S8.

**TABLE 1:** Maximum intracellular mannitol accumulation expressed on a biomass basis and as volumetric concentration during salinity stress.

Species	Salinity (g L^-1^)	Maximum Mannitol (mg g^-1^ DW)	Biomass (g L^-1^)	Mannitol (mg L^-1^ culture)
*Dunaliella tertiolecta*	60	28	0.56	15.68
*Dunaliella tertiolecta*	80	16	0.53	8.48
*Nannochloropsis oculata*	60	34	0.52	17.68
*Nannochloropsis oculata*	80	27	0.60	16.20

The comparative responses summarized in Table 2 demonstrate that salinity affected multiple aspects of cellular metabolism in a species-specific manner. While the freshwater species responded primarily through changes in carbohydrate, protein, and pigment composition without detectable polyol accumulation, the marine species exhibited intracellular mannitol accumulation together with distinct shifts in biomass composition. A common response across all four species was an initial decline in chlorophyll following salinity stress, although the extent of recovery differed among species. The magnitude and persistence of chlorophyll decline differed among species. Similar transient reductions in chlorophyll under salt stress have been reported previously and have been attributed to salt-induced photoinhibition, whereby ionic and osmotic stress impair photosystem stability and temporarily reduce photosynthetic efficiency (Ji et al., 2018; McLachlan, 1961; Tammam et al., 2011; Yun et al., 2019). The concurrent retention or accumulation of carotenoids in the marine species suggests that photoprotective mechanisms remain active despite chlorophyll depletion.

**TABLE 2:** A summary of key physiological and metabolic responses to salinity stress. Key physiological and biochemical responses observed in this study are summarized to facilitate cross-species comparison and interpretation.

Species	Polyol production	Dominant polyol	Highest mannitol yield (mg g^-1^ DW)	Carbohydrate response	Protein response	Pigment response	Observed stress response	Potential application
Freshwater species								
*Chlorella vulgaris*	Not detected	—	—	Decreased under stress	Increased in control/moderate salinity; decreased at high salinity	Chlorophyll ↓, carotenoids relatively stable	Non-polyol osmoadaptation Continued nitrate assimilation; no detectable polyols	Lipid-oriented cultivation (reported)- not evaluated in this study
*Scenedesmus obliquus*	Not detected	—	—	Increased in stressed cultures	Significant decline at high salinity	Chlorophyll ↓, carotenoids partially recover	Increased carbohydrates; reduced protein at high salinity	Carbohydrates accumulation
Marine species								
*Dunaliella tertiolecta*	Yes	Mannitol	28	Increased up to 50% DW at 60 g L^-1^	Initial decline, partial recovery	Carotenoids ↑, chlorophyll recovers	Mannitol accumulation	Polyols
*Nannochloropsis oculata*	Yes	Mannitol	34	Moderate increase, then decrease	Proteins ↓	Carotenoids ↑	Mannitol accumulation	Intracellular mannitol with higher volumetric concentration

### 3.6 Nitrogen assimilation under balanced and unbalanced growth

In this study, balanced growth refers to conditions where nitrogen assimilation and biomass production remain closely coupled. In contrast, unbalanced growth describes stress conditions under which this relationship becomes uncoupled, resulting in altered biomass composition and metabolite accumulation. Nitrogen yield analysis revealed contrasting responses between marine and freshwater species under salinity stress. During the pre-stress phase, both *Dunaliella tertiolecta* (Figure 8a) and *Nannochloropsis oculata* (Figure 8b) exhibited comparable nitrate yields (0.030–0.036 g N g^-1^ DW), indicating balanced growth conditions in which nitrogen assimilation was closely coupled to biomass production (Baroukh et al., 2014; Raven and Geider, 2003).

**FIGURE 8:**
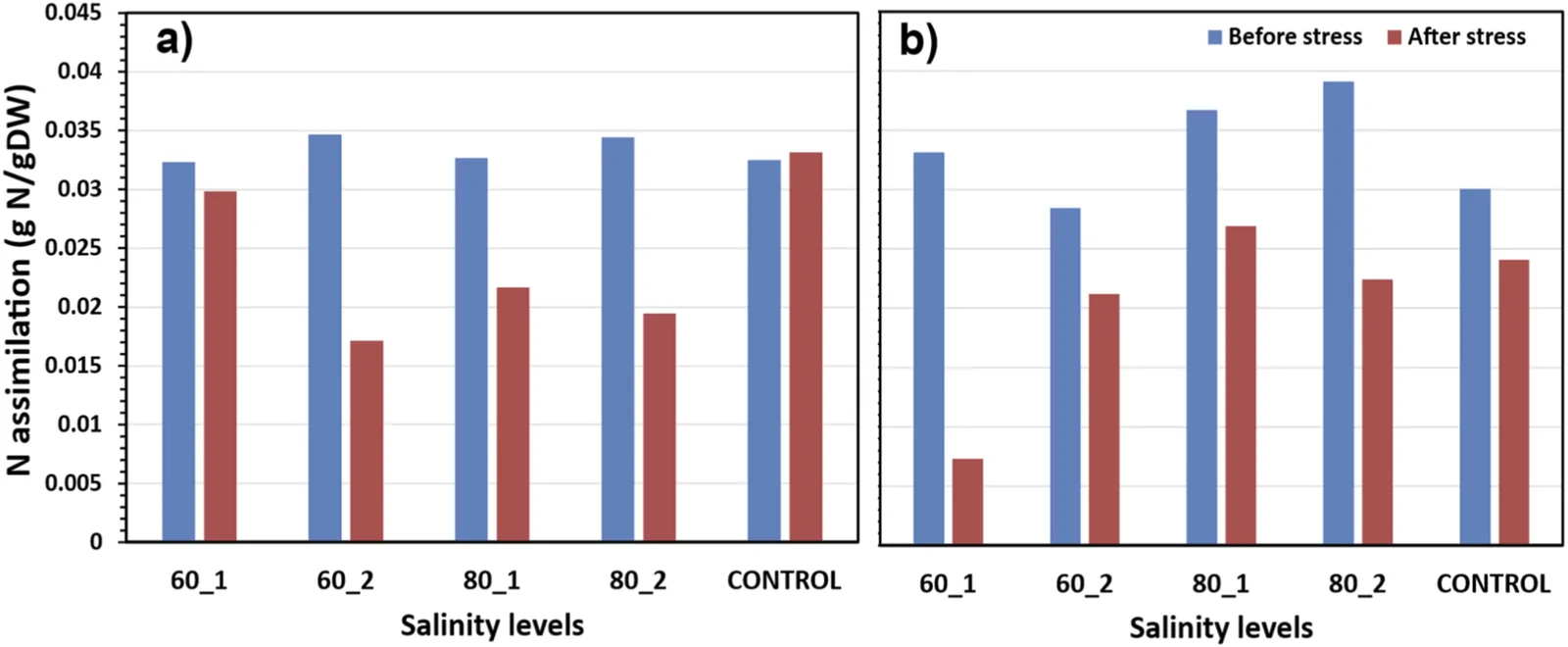
Nitrogen assimilated (yield) per biomass before and after stress: **(a)** *Dunaliella tertiolecta* **(b)** *Nannochloropsis oculata*. Values represent cultures grown at 60 and 80 g L^-1^ salinity (in duplicates). Control cultures were maintained under non-stress conditions.

Salinity stress was imposed after all cultures reached exponential growth under identical cultivation conditions, thereby minimizing differences in biomass at the onset of the stress phase. No additional nutrients were supplied during the subsequent 72 h stress period. Therefore, the observed physiological responses primarily reflect the effects of salinity rather than renewed nutrient supplementation. Although nitrate availability gradually declined during the stress phase, the short duration of the experiment allowed the evaluation of early stress responses before complete nitrogen depletion occurred.

Following salinity stress, both marine species exhibited reduced nitrate assimilation yield, indicating a transition toward unbalanced growth. The decline was most pronounced in *D. tertiolecta*, particularly under 80 g L^-1^ salinity, where nitrate yield decreased to approximately 0.02 g N g^-1^ DW. In contrast, *N. oculata* maintained intermediate levels of nitrogen assimilation despite stress, suggesting partial retention of nitrogen metabolism.

The reduced nitrate assimilation observed in marine species coincided with the accumulation of carbohydrates, polyols, and carotenoids, was associated with changes in biomass composition during salinity stress. Such responses are consistent with previous reports of increased accumulation of stress-associated metabolites (Raven and Geider, 2003).

In Chlorella (Figure 9a) and Scenedesmus (Figure 9b), the nitrate yield per unit biomass increased following the onset of stress, in contrast to *Dunaliella*, where nitrogen assimilation declined. This suggests that nitrogen assimilation remained active despite reduced growth. Although the fate of assimilated nitrogen was not directly investigated, the results indicate that freshwater species employ a distinct stress-response strategy compared with marine species.

**FIGURE 9:**
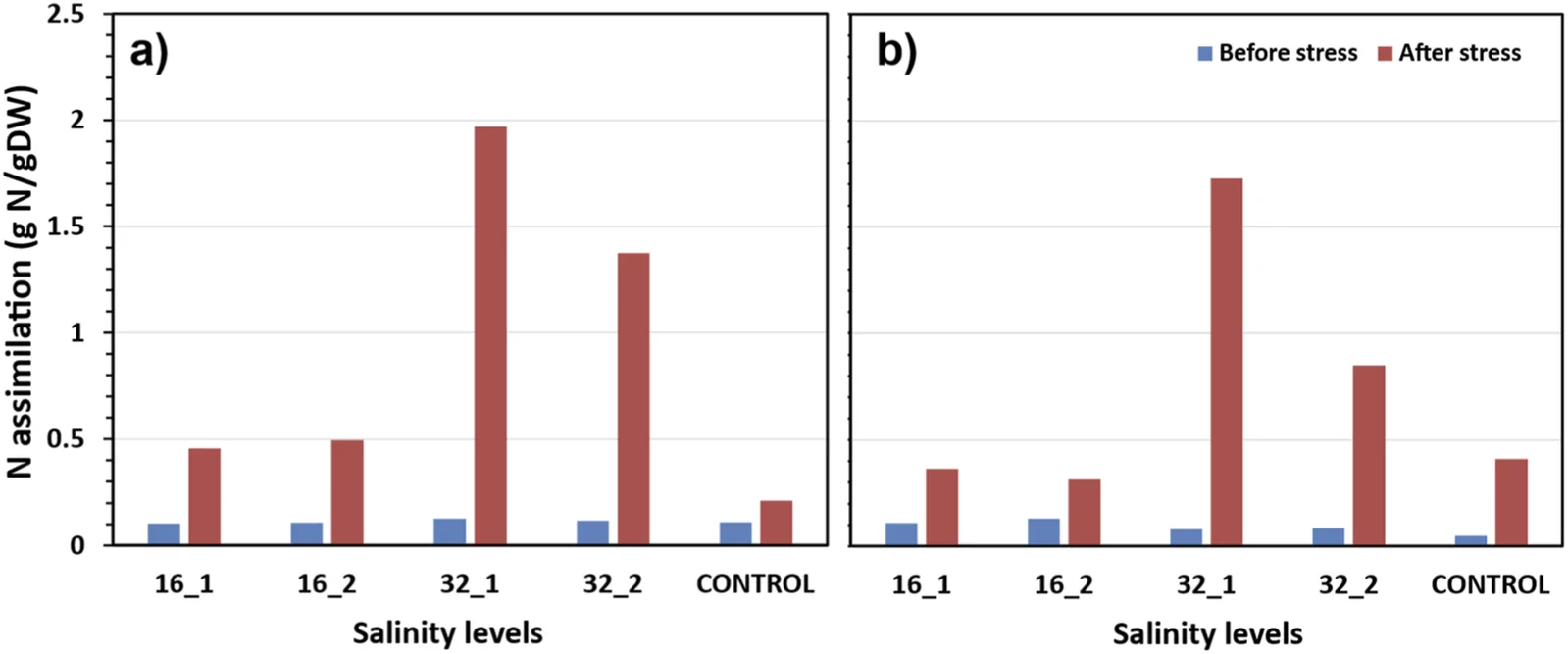
Nitrogen assimilated (yield) per biomass before and after stress to understand balanced and unbalanced growth: **(a)** *Chlorella vulgaris* **(b)** *Scenedesmus obliquus*. Values represent cultures grown at 16 and 32 g L^-1^ salinity (in duplicates). Control cultures were maintained under non-stress conditions.

Because nitrogen assimilation was expressed relative to biomass, and BG11 contained substantially higher initial nitrate-N concentrations than F/2 medium, absolute nitrogen-yield values should not be directly compared between freshwater and marine species. Nevertheless, the contrasting trends within each cultivation medium were consistent, with the marine species exhibiting reduced nitrogen assimilation following stress, whereas the freshwater species maintained comparatively higher values. Overall, the contrasting nitrogen assimilation patterns observed between freshwater and marine microalgae provide additional insight into the species-specific metabolic responses identified in this study. In this screening, the two species exhibiting intracellular mannitol accumulation also showed reduced nitrogen assimilation yield after stress, whereas the two species without detectable polyol accumulation showed increased or comparatively maintained yields.

## 4 Conclusion

This study systematically compared freshwater and marine microalgae for intracellular polyol accumulation under salinity stress using a two-stage cultivation strategy. Among the four species evaluated, mannitol was the dominant polyol detected and intracellular mannitol accumulation was detected exclusively in the marine microalgae *Dunaliella tertiolecta* and *Nannochloropsis oculata*, whereas the freshwater species *Chlorella vulgaris* and *Scenedesmus obliquus* exhibited alternative stress responses involving changes in carbohydrate, protein, and pigment composition without detectable polyol accumulation. These findings demonstrate that intracellular mannitol accumulation is a species-specific response rather than a universal adaptation to salinity stress.

Moderate salinity (60 g L^-1^) promoted the highest intracellular mannitol accumulation in both marine species, with *N. oculata* achieving the greatest biomass-specific (34 mg g^-1^ DW) and volumetric (17.68 mg L^-1^ culture) concentrations. The concurrent changes observed in carbohydrates, proteins, pigments, and nitrogen assimilation further highlight distinct metabolic responses among freshwater and marine microalgae under comparable cultivation and stress-assessment procedure.

This study represents a comparative screening investigation conducted under controlled laboratory conditions and therefore serves as a foundation for future process development rather than demonstrating an optimized production platform. The exclusive focus on intracellular polyols, together with the absence of direct measurements of extracellular glycerol, oxidative stress markers, enzyme activities, and transcriptional responses, limits mechanistic interpretation of the observed metabolic responses.

Future studies should optimize salinity, light, nutrient availability, and stress duration to enhance intracellular polyol accumulation while maintaining biomass productivity. Integrated biochemical and molecular analyses will also be required to clarify the regulation of polyol biosynthesis.

## Data Availability

The original contributions presented in the study are included in the article/Supplementary Material, further inquiries can be directed to the corresponding authors.
