# Supplementary material for: A comparative screening of freshwater and marine microalgae for salinity-induced intracellular mannitol accumulation
Source: Front Bioeng Biotechnol. 2026 Sep 17;14:1922237. doi: 10.3389/fbioe.2026.1922237 (PMC13627334; doi:10.3389/fbioe.2026.1922237)
Supplement: Supplementary file 1 [file Supplementaryfile1.docx]

**Supplementary material
A comparative screening of freshwater and marine microalgae for salinity-induced intracellular mannitol accumulation**

Shibin Nadersha^1,2^, Ashfaq Ahmad^3,4^, Syed Salman Ashraf^3,4^ and Jorge Rodríguez^1^

^1^ Department of Chemical and P. Engineering. Khalifa University PO Box 127788, Abu Dhabi, UAE

^2^ Research and Innovation Centre on CO_2_ and H_2_ (RICH), Khalifa University PO Box 127788, Abu Dhabi, UAE

^3^ Department of Biological Sciences. Khalifa University PO Box 127788, Abu Dhabi, UAE

^4^ Center for Biotechnology, Khalifa University PO Box 127788, Abu Dhabi, UAE

### 1. F/2 media composition

**Table S1:** Nutrient composition of F/2 medium used for algal cultivation in this study

| Stock Solutions | Component | Concentration (g/L) |
| --- | --- | --- |
| Nitrogen source | NaNO_3_ | 75 |
| Phosphorous source | NaH_2_PO_4_.2H_2_O | 5.65 |
| Trace Metal Stock | Na_2_EDTA | 4.16 |
|  | FeCl_3_.6H_2_O | 3.15 |
|  | CuSO_4_.5H_2_O | 0.01 |
|  | ZnSO_4_.7H_2_O | 0.022 |
|  | CoCl_2_.6H_2_O | 0.01 |
|  | MnCl_2_.4H_2_O | 0.18 |
|  | Na_2_MoO_4_.2H_2_O | 0.006 |
| Vitamin Mix | Vitamin B_12_ (Cyanocobalamin) | 0.0005 |
|  | Vitamin B_1_ (Thiamine Hydrochloride) | 0.1 |
|  | Biotin | 0.0005 |

### 2. BG11 media compositision

**Table S2**: Detailed composition of BG11 culture medium used in this study.

| Component | Stock solution concentration (g/L) | Amount mL/L |
| --- | --- | --- |
| NaNO3 | 37.5 | 10 |
| KH2PO4 | 1 | 10 |
| MgSO4·7H2O | 1.875 | 10 |
| CaCl2·2H2O | 0.9 | 10 |
| Citric acid·H2O | 0.15 | 10 |
| Ferric ammonium citrate | 0.15 | 10 |
| Na2EDTA·2H2O | 0.025 | 10 |
| Na2CO3 | 0.5 | 10 |
| BG11 Trace Metal Solution | - | 1 |

**Table S3:** Composition of trace metal solution for BG11 media

| Component | Concentration (mg/L) |
| --- | --- |
| H_3_BO_3_ | 2.86 |
| MnCl2·4H2O | 1.81 |
| ZnSO4·7H2O | 0.22 |
| Na2MoO4·2H2O | 0.39 |
| CuSO4·5H2O | 0.08 |
| Co(NO3)2·6H2O | 0.05 |

### 3. Preparation of artificial seawater

Instant Ocean® Sea Salt (Instant Ocean, Aquarium Systems Inc., France) was used to prepare the synthetic saltwater that makes up the majority of the F/2 medium. After adding the salts to deionised water, any remaining salts were filtered out of the mixture using a Whatman™ filter (Ø 150 mm, pore size 2.5 μm). Using a Multi-Parameter Meter (HQ40d, HACH®, USA), the salinity was adjusted to 32 g L-1 TDS following filtering.

### 4. HPLC–ELSD operating conditions for polyol analysis

**Table S4**: Instrumental and chromatographic conditions used for polyol analysis by HPLC–ELSD

| LC system | Waters Alliance HPLC System |
| --- | --- |
| Detector | ELSD |
| Vials | LCGC Certified Clear Glass, Max Recovery, with  Cap and Preslit PTFE/Silicone Septum, 1.5 mL  (p/n: 186000327C) |
| Filter | Syringe Filter 0.2 µm PVDF (p/n: WAT200806) |
| Columns | Atlantis Premier BEH Z-HILIC, 4.6 x 150 mm, 2.5  µm (p/n: 186009994) |
| Column temperature | 55 °C |
| Sample temperature | 25 °C |
| Detector temperature | 55 °C |
| Injection volume | 5 µL |
| Flow rate | 0.4 mL/min |
| Mobile phase | 5-95% Acetonitrile:water gradient method |
| Sample diluent | 50:50 Acetonitrile:water |
| Seal wash | 5:95 Acetonitrile:water |
| Needle wash: | 5:95 Acetonitrile:water |
| Purge solvent | 50:50 Acetonitrile:water |

**Figure S1**: **Representative HPLC–ELSD chromatogram of polyol standards used for qualitative identification and quantification.** Peaks correspond to glycerol (21.7 min), xylitol (26.1 min), sorbitol (26.7 min), mannitol (29.8 min), and inositol (34.2 min). Peak identities were confirmed using authentic standards, and quantitative analysis was performed using external calibration curves.

**Table S5**: Calibration characteristics of polyol standards used for HPLC–ELSD quantification

| **Polyol** | **Calibration range (mg mL⁻¹)** | **Calibration model** | **R²** |
| --- | --- | --- | --- |
| Mannitol | 0.05–2.00 | Quadratic | 0.9936 |
| Sorbitol | 0.05–2.00 | Quadratic | 0.9934 |
| Glycerol | 0.05–2.00 | Quadratic | 0.9937 |
| Xylitol | 0.05–2.00 | Quadratic | 0.9946 |
| Arabitol | 0.05–2.00 | Quadratic | 0.9949 |
| Inositol | 0.05–2.00 | Quadratic | 0.9913 |

External calibration was performed using mixed polyol standards prepared at concentrations of 0.05, 0.10, 0.20, 0.40, 0.60, 0.80, 1.00 and 2.00 mg mL⁻¹. Calibration curves were fitted using quadratic regression to account for the nonlinear response of the ELSD detector. The analytical method exhibited a limit of detection (LOD) of 0.05 mg mL⁻¹.
